# Plant secondary metabolites exert therapeutic effects by modulating autophagy pathways

**DOI:** 10.3389/fpls.2026.1782554

**Published:** 2026-03-27

**Authors:** Yinuo Ma, Yongxia Cui, Weiming Hu, Qi Zhang, Xiaoqing Zhou, Fen Liu

**Affiliations:** 1 Basic Medical Experiment Center, School of Traditional Chinese Medicine, Jiangxi University of Chinese Medicine, Nanchang, China; 2 Jiangxi Provincial Key Laboratory of Plant Germplasm Resources Innovation and Genetic Improvement, Lushan Botanical Garden, Jiangxi Province and Chinese Academy of Sciences, Jiujiang, China; 3 Department of Oncology, The Affiliated Hospital of Southwest Medical University, Luzhou, Sichuan, China; 4 Jiangxi Academy of Forestry, Nanchang, China

**Keywords:** autophagy, drugs of plant origin, human health, pharmacological properties, plant secondary metabolites

## Abstract

Modern research indicates that plant secondary metabolites possess pharmacological properties such as anti-inflammatory, antioxidant and antitumor effects. Most plant secondary metabolites hold significant research value for the treatment of human diseases. Current research extensively explores the potential mechanisms through which plant secondary metabolites exert therapeutic effects against various diseases, with one of the primary pathways involving their modulation of autophagy in relevant human cells and organelles, thereby inhibiting disease progression This review summarizes several categories of different plant secondary metabolites-including flavonoids, anthraquinones, coumarins and saponins-and elucidates their mechanisms in treating various diseases through autophagy regulation. This information contributes to the development and application of herbal medicines.

## 1 Introduction

Autophagy, as a pathway regulating cellular degradation together with proteasome-mediated degradation, constitutes the main routes for protein and organelle turnover. These degradation pathways are particularly important during development and under certain environmental stress conditions such as during starvation (Xiong et al., 2025), cell differentiation (Vidyawan et al., 2025), cell death (Young et al., 2012) and aging (Kang et al., 2015).

Autophagy has three types: macroautophagy, microautophagy and chaperone-mediated autophagy. Macroautophagy is an evolutionarily conserved process that mainly forms autophagosomes (a double-membrane vesicle) to selectively or non-selectively transport proteins, damaged organelles, pathogens, and other intracellular contents to lysosomes for degradation and recycling thereby maintaining cellular homeostasis (Klionsky and Emr, 2000; McCarty, 2022). Selective macroautophagy can be achieved through various macroautophagy adaptors/receptors that specifically recognize target proteins, thereby completing the autophagic process (Gatica et al., 2018). The process of microautophagy is a non-selective lysosomal degradation mechanism. Its primary function involves the invagination of organelle membranes within the lysosomal system leading to their internalization. Subsequently these membranes are sealed into small vesicles and detached from the membrane for intraluminal degradation (Li et al., 2012; Schuck, 2020; Krause and Cuervo, 2021). The chaperone-mediated autophagy (CMA) involves the utilization of molecular chaperone complexes to recognize cytosolic proteins that possess specific peptide sequence motifs. These proteins are then delivered to lysosomes where they are exclusively degraded as the sole cargo of this pathway. Only those proteins containing a particular targeting motif within their sequences can be degraded through this mechanism (Majeski and Dice, 2004; Kaushik and Cuervo, 2018). The purpose of autophagy is not merely to eliminate substances rather it functions as a dynamic cyclical system that generates new building blocks and energy for cellular renewal and homeostasis (Mizushima and Komatsu, 2011).

Autophagy was once considered a non-selective mechanism. However recent research has demonstrated that it can specifically target and remove unwanted or detrimental cytoplasmic components and damaged materials. This process primarily serves a protective role for cells and necessitates precise regulation to accurately respond to various stimuli encountered by cells, allowing them to adapt to their ever-changing environment (Dikic and Elazar, 2018). In plants, adaptive cellular homeostasis is strongly supported by secondary metabolites, which function as a chemically diverse defensive and signaling arsenal enabling plants to survive biotic and abiotic stresses, a concept often described as nature’s ‘gifted arsenal’ for plant resilience (Ahmed et al., 2024).

In recent years, studies have found that many plant secondary metabolites (PSMs)possess the ability to regulate autophagy and, by precisely modulating the autophagic process, have demonstrated significant therapeutic potential in various disease models.

PSMs are considered ideal candidates for the development of autophagy-modulating therapeutics due to their multi-target effects and relatively low toxicity. PSMs exert their functions by targeting core signaling nodes for autophagy initiation. Studies have shown that they can enhance autophagic activity in neuronal cells by activating MAP2K2 and PLK1 kinases (Chen et al., 2017), promoting the clearance of toxic protein aggregates—a mechanism that offers potential therapeutic strategies for Alzheimer’s disease and Parkinson’s disease. During the stages of autophagosome formation and expansion, PSMs can promote the ATG8-PE conjugation system and enhance autophagosomal membrane stability (Popelka and Klionsky, 2021), thereby exhibiting effects in extending lifespan and reducing age-related cellular de-cline in yeast, nematodes, and cultured human cells.

The regulatory effect of PSMs on specific types of autophagy also demonstrates the precision and specificity of their mechanisms of action. Plant secondary metabolites promote the clearance of damaged mitochondria containing excess Fe²^+^ through mitophagy by activating the Nrf2 pathway (Liu et al., 2025), while simultaneously upregulating the expression of antioxidant proteins such as GPX4 and SLC7A11 to inhibit the occurrence of ferroptosis.

The ability of PSMs to intervene in disease progression by regulating the autophagic process is closely related to their unique chemical structures and biological activities. These compounds modulate different stages of autophagic flux by targeting key signaling pathways such as mTOR, Nrf2, NF-κB, and AMPK, thereby playing important roles in the treatment of neurodegenerative diseases, cardiovascular diseases, cancer, and metabolic disorders.

## 2 Autophagy and disease treatment

Autophagy is widely regarded as a double-edged sword with unique roles in tumor suppression and growth promotion. Autophagy can inhibit the occurrence of cancer by eliminating oncogenic, unfolded or aggregated proteins (Li et al., 2025) and damaged organelles. In plants, autophagy is not only activated under nutrient-deficient conditions to recycle cellular materials but also widely participates in growth (Lv et al., 2024), development (Zhang et al., 2025), immune responses (He et al., 2025), and adaptation to various stress conditions (Yu et al., 2025). Under specific circumstances, autophagy may also be involved in executing programmed cell death, and its overactivation can even cause damage to the plant (Chen et al., 2009). At the same time, autophagy also plays a certain role in controlling tumor development by maintaining genomic stability, eliminating endogenous reactive oxygen species (ROS) sources, eliminating oncogenic proteins and inducing immune responses. In contrast, autophagy can also promote tumor metabolism and cancer cell survival through metabolite recycling and serve as a cellular protection mechanism for cancer cells in response to anticancer therapy (Kenific and Debnath, 2015). This protective mechanism is mainly manifested in the fact that when tumor is formed, tumor cells produce metabolites conducive to survival through autophagy when facing survival pressures such as hypoxia (Yuan et al., 2025) or oxidative (Drago-Garcia et al., 2025) stress, helping tumor cells survive and metastasize. In addition, most studies believe that autophagy of cancer cells can promote the proliferation of cancer cells and the emergence of drug resistance, which poses a great challenge to the prognosis of cancer patients (Gao et al., 2025). For example, in the development of breast cancer, Chromosome 1 Open Reading Frame 116 (C1ORF116) act as an autophagy receptor to induce autophagy, providing redox homeostasis for dormant cells, which greatly improves the survival rate of dormant breast cancer cells (Drago-Garcia et al., 2025).

Autophagy plays a dynamic role in the occurrence and progression of tumors, which determines that therapeutic strategies based on autophagy regulation must take tumor stage into account. In the early stages of tumorigenesis, autophagy exerts a tumor-suppressive role by clearing damaged organelles and maintaining genomic stability. At this stage, activating autophagy is suggested to help prevent tumor occurrence. In the advanced stages of tumor progression, established tumor cells often exploit autophagy to resist hypoxia, nutrient deprivation, and chemotherapeutic stress, shifting autophagy into a tumor survival mechanism. At this stage, inhibiting autophagy becomes the therapeutic strategy.

Autophagy also plays a non-negligible role in other diseases, for example, current studies have found that autophagy directly affects the occurrence and development of neurodegenerative diseases. Abnormal protein accumulation in nerve cells is a hallmark of several neurodegenerative diseases and is directly linked to impaired macroautophagy and CMA. Neuronal cells remove damaged mitochondria, toxic proteins, or microaggregates through autophagy, which contributes to preventing the occurrence of neurodegenerative diseases (Yang and Klionsky, 2020). Dysfunctional autophagy may lead to neurodegenerative diseases through abnormal inflammatory signaling (Sliter et al., 2018). For instance, it has been found that one of the causative factors of Alzheimer’s disease (AD) is mainly the excessive accumulation of amyloid β-protein (Aβ), and the autophagic activity of nerve cells can effectively degrade Aβ (Wang et al., 2025). Therefore, finding targets for activation of related autophagy pathways may be one of the strategies for the prevention and treatment of AD in the future. However, under the stress of hypoxic-ischemic environment, the excessive autophagic reaction of nerve cells will cause secondary damage to nerve cells. Research has indicated that the activation of the AKT-FOXO4 pathway helps to reduce the overexpression of autophagy in nerve cells under ischemic and hypoxic conditions, thereby protecting the nerves (Shen et al., 2025). Consequently, in terms of disease development, the deterioration of diseases caused by uncontrolled autophagy is also one of the difficulties that need to be overcome in the current disease treatment.

Collectively, current studies have shown that autophagy plays an important role in the mammalian oxidative stress response and its interrelationship with other stress defense systems (Ornatowski et al., 2020).

## 3 Plant secondary metabolites treat autophagy related diseases

Plant secondary metabolites (PSMs), natural compounds derived from plants, exhibit a variety of pharmacological effects including anti-inflammatory, anti-tumor, and antioxidant activities. They have demonstrated exceptional potential in treating human diseases, particularly those related to autophagy (Yeshi et al., 2022).

According to the chemical structure and biosynthesis pathway, Based on their chemical structures, PSMs can be divided into the following five main categories: Phenolics, Terpenoids, nitrogen-containing compounds, Carbohydrates. PSMs are widely used in the treatment of human diseases. This review selects seven representative categories of compounds—flavonoids, anthraquinones, coumarins, tannins, saponins, alkaloids, and polysaccharides—for in-depth discussion.

According to the chemical structure and biosynthesis pathway, PSMs can be divided into flavonoids, anthraquinone, coumarins, saponins, tannins, alkaloids and polysaccharides. Flavonoids are widely found in fruits, vegetables, and medicinal plants (Liu et al., 2018), and have the basic skeletal structure of C6-C3-C6 (Farooq and Ngaini, 2025), including subtypes such as chalcone, nobiletin, and flavanone. The basic parent nucleus of anthraquinone compounds is anthraquinone, and there are often substituents such as hydroxyl, methyl, and carboxyl groups on the parent nucleus, which often determine the medicinal properties of quinone anthracene derivatives (Ge et al., 2019). Alkaloids are nitrogenous basic compounds, which are mainly found in medicinal plants and have significant biological activity (Jiang et al., 2025). Coumarin is a naturally occurring benzopyranone compound found in plants, featuring a unique oxygen-containing heterocyclic structure. This structure makes coumarin a commonly used natural scaffold in drug design (Abdelaal et al., 2025). Various coumarin hybrid derivatives have demonstrated excellent drug properties in the treatment of various diseases (Valencia et al., 2025). Saponins are a class of glycosides whose aglycones are triterpenoids or steroidal compounds, mainly distributed in higher plants and marine organisms (Kulkarni et al., 2025). Tannins are a type of polymer molecule with a hydrophobic aromatic ring core surrounded by hydroxyl groups (Cheng et al., 2025). The biological effects of tannins mainly depend on the grade of polymerization of their chemical structure. Polysaccharides are usually natural macromolecular polymers consisting of more than ten monosaccharides linked together and are one of the indispensable compounds in cellular life activities, the polysaccharides from natural sources have unique biological activities and are important for research in the biomedical field (Sun et al., 2025).

The ability of these PSMs to intervene in disease progression by regulating the autophagy process is closely related to their unique chemical structure and biological activity. For example, flavonoids often have a polyphenol structure that gives them antioxidant and signal-regulating functions (Pu et al., 2025). It is worth noting that PSMs can regulate the occurrence and development of cellular autophagic activities by regulating various signaling pathways, which provides important implications for the development of autophagy modulators based on these natural products (**Table 1**).

**Table 1:** Classification of major plant secondary metabolites and their autophagy regulatory mechanisms.

Category	Representative compounds	Direction of autophagy modulation (Evidence level)	Major autophagy regulatory mechanisms	Associated diseases
Flavonoid	Tricetin	Cytoprotective au-tophagy↑(mice)	Regulate PI3K/AKT/mTOR The signaling pathway promotes autophagy in neuronal cells, reduces Aβ deposition, and inhibits Tau protein phosphorylation	Neurodegenerative diseases (Wu et al., 2025)
Anthraquinone	Emodin	Protective mitophagy↑ (cell and mice)	Promote mitophagy, maintain mitochondrial homeostasis, and inhibit inflammasome activation	Inflammatory diseases (Fu et al., 2025)
Coumarin	Umbelliferone	Cytotoxic autophagy↓ (cell and mice)	Inhibition of nephrolithiasis formation by stimulating autophagy induced by the PI3K/AKT pathway	Kidney damage (Xu et al., 2025)
Saponin	20(R)-ginsenoside Rg3	Cytotoxic autophagy↓ (mice)	Activation of the PI3K/AKT/mTOR signaling pathway inhibits autophagy and prevents cerebral ischemia-reperfusion injury	Cardiovascular and cerebrovascular diseases (Tao et al., 2025)
Alkaloid	Nuciferine	Protective mitophagy↑ (mice)	Inhibition of the PI3K/Akt/mTOR signaling pathway promotes autophagy and improves stage IV renal fibrosis.	Diabetic nephropathy (Li et al., 2025)
Tannins	Punicalagin	Cytotoxic autophagy↓ (mice)	Activate the AKT-FOXO4 pathway, inhibit the overexpression of autophagy in nerve cells, and exert neuroprotective effects	hypoxic-ischemic encephalopathy (Shen et al., 2025)
Polysaccharide	Lycium barbarum polysaccharide	Protective mitophagy↑ (cell)	Activates AMPK/PINK1/Parkin-mediated mitophagy to repair mitochondrial function and promote the breakdown of glucose and lipids	Sarcopenic obesity (Ren et al., 2025)

↑ indicates promotion, ↓ indicates inhibition Various previous studies have shown that PSMs can modulate the autophagic activities of human cells, thereby influencing human health. In this review, the following representative categories of PSMs were selected, their roles in regulating cellular autophagic activities were summarized, and their mechanisms of action in influencing cellular vital activities through the modulation of autophagy were described.

### 3.1 Phenolics

Plant-derived phenolic compounds are a class of structurally diverse secondary metabolites widely found in the plant kingdom. The polyhydroxy substitution of the benzene ring is their most prominent chemical feature. This review selects representative phenolic compounds, including flavonoid, anthraquinone, coumarin and tannin, for discussion.

#### 3.1.1 Flavonoid

Flavonoids are widely found in a variety of plants. Daily consumption of vegetables fruits and plant-derived beverages are the main dietary sources of flavonoids. Flavonoids all have an identical flavonoid skeleton-a 15-carbon phenylpropane chain. Based on the differences in their chemical structures, flavonoids are further classified into six major groups: isoflavones, flavanones, flavanols, flavonols, flavonoids, and anthocyanins (Panche et al., 2016).

Current pharmacological studies have proved that flavonoids have pharmacological effects such as antioxidant (Su et al., 2025), antitumor (Hao et al., 2025), anti-inflammatory (Kuwar and Kalia, 2025), neuroprotective and cardiovascular protection. In cancer therapy, flavonoids can inhibit the proliferation and spread of many common cancer cells by inducing cell autophagy. Currently, the anticancer effects of plant-derived flavonoids and their mechanisms are still under research, which may provide new drug choices for future cancer therapy (**Table 2**). Beyond cytoprotective roles, flavonoids can drive autophagy-associated apoptosis, as demonstrated for luteolin, which induces mitochondrial depolarization, caspase activation, and programmed cell death in breast cancer models, highlighting coordinated regulation between autophagic flux and apoptotic execution (Ahmed et al., 2019a).

**Table 2:** The mechanism of plant-derived flavonoids in treating cancers.

Cancer type/model	Material	Type of study	Affect	Mechanism	Key indicators	Ref.
hepatocellular carcinoma	Icaritin	*in vitro* experiment (cell)	Regulation of mitochondrial autophagy induction of apoptosis	mitochondrial damage, Reactive oxygen species (ROS) accumulation (protective autophagy)	PINK1-Parkin signaling-mediated mitochondrial autophagy↑	(Luo et al., 2024)
Human Oral Squamous Cell Carcinoma	Latifolin	*in vitro* experiment (cell)	Induction of autophagy and apoptosis	Anti-metastatic activity by inactivating focal adhesion kinase (FAK)/non-receptor tyrosine kinase (Src) effectively preventing cell migration invasion and adhesion. PI3K/AKT/mTOR/p70S6K signaling induces apoptosis. (Cytotoxicity)	dephosphorylating necroptosis-regulatory proteins (RIP1 RIP3 and MLKL) autophagic-related proteins and autophagosome formation↓	(Yun et al., 2022)
human colon cancer	Myricetin	*in vitro* experiment (cell)	Reduces cell viability and induces autophagy and apoptosis	Inhibition of PI3K/Akt/mTOR signaling pathway induces apoptosis and autophagy in cancer cells. (protective autophagy)	Increased expression of LC3-II/β-actin and Beclin-1/β-actin in HCT116 and SW620 cells↑.	(Zhu et al., 2020)
non-small cell lung cancer	Baicalin	*in vitro* experiment/*in vivo* experiment (cell and mice)	Activation of membrane cation channels of lysosomes is used to block autophagic fluxes. Inhibition of cancer cell proliferation	The cytoplasmic Ca^2+^ imbalance depolarization of mitochondrial membrane potentials and ROS accumulation in NSCLC cells. (Cytotoxicity)	dysfunction in lysosomal pH elevation↓.	(Dong et al., 2024)
Human mucoepidermoid carcinoma	Chrysin	*in vitro* experiment (cell)	Reduces cell viability and induces autophagy and apoptosis	MAPK/ERK pathway induces apoptosis in cancer cells. Exerts cytoprotective effects through the ERK/mTOR pathway. (protective autophagy)	ERK1/2a, key protein of the MAPK pathway is reduced. Increased P-JNK and P-p38. Bax/Bcl-2 ratio and c-PARP levels were increased. ↑	(Jung et al., 2022)

↑ indicates autophagy promotion, ↓ indicates autophagy inhibition In the early stages of tumorigenesis, autophagy suppresses tumor development by facilitating the clearance of damaged organelles and maintaining genomic stability. During this phase, flavonoids activate autophagy, contributing to the prevention of tumor formation. Studies have shown that flavonoids exert protective effects on healthy or moderately damaged cells, while exhibiting toxic effects on tumor or pre-cancerous cells. This selective cytotoxicity confers unique advantages upon flavonoids in cancer chemoprevention. In the advanced stages of tumor progression, simply activating autophagy may promote tumor survival, while inhibiting autophagy is a more rational therapeutic strategy (D’Arcy, 2022).

Long-term use of flavonoids may lead to the adaptive upregulation of basal autophagy levels in cancer cells, forming a drug resistance mechanism. Tumor cells adaptively upregulate autophagy-related genes (such as ATG5, ATG7, Beclin-1, etc.) to enhance autophagic flux, thereby counteracting the cytotoxicity of flavonoids (Zhu et al., 2021). Tumor cells can restore the activity of the PI3K/AKT/mTOR pathway through bypass activation, thereby evading the autophagy regulation by PSMs.

Nobiletin from *Citrus nobilis* Lour can activateSIRT-1/FOXO3a-mediated cellular autophagy and thereby ameliorates hepatic ischemia and reperfusion injury by activating the expression of proteins that control cellular autophagy as well as mitochondrial dynamics (Dusabimana et al., 2019). *Bauhinia championii* flavonoids inhibit cardiomyocyte autophagy and apoptosis through PI3K/AKT activation thereby attenuating myocardial ischemia/reperfusion injury in rats (Jian et al., 2015). The reduction of myocardial ischemia/reperfusion loss is important for the prevention and treatment of ischemic heart disease and the flavonoid-induced cardiac protection mechanism has clinical significance for the development of drugs for ischemic heart disease and may provide a new option for the treatment of cardiovascular diseases. In ischemic cardiac injury, phytochemicals such as curcumin modulate AMPK-mTOR-dependent autophagy, contributing to mitochondrial preservation, attenuation of oxidative stress, and improved cardiomyocyte survival, underscoring disease-specific roles of autophagy beyond oncology (Ahmed et al., 2019b).

There is also some research potential for flavonoids in neuroprotection. For example, luteolin can inhibit mitochondrial hyperautophagy and reduce lysosomal activity by inactivating BNIP3L/NIX and activate mTORC1 to attenuate glutamate accumulation-induced autophagy and apoptosis in neuronal cells (Vongthip et al., 2024). This study demonstrates that flavonoids may treat neurodegenerative diseases by modulating autophagy and mitochondrial dynamics in neuronal cells.

Flavonoids are currently recognized as effective antioxidants that can scavenge free radicals and ROS (Wang et al., 2020), inhibit the activity of free radical synthesizing enzymes, and increase the intracellular concentrations of glutathione (GSH) and antioxidant molecules (Cotelle, 2001).They play a crucial role in inhibiting cell cycle progression and inducing autophagy regulation in cells (Liu et al., 2018). The compound Nobiletin has demonstrated significant effects in antioxidant activity and anti-apoptosis. Previous studies have indicated that Nobiletin can ameliorate oxidative stress responses induced by AD and liver ischemia-reperfusion injury through the induction of autophagy (Dusabimana et al., 2019). Nobiletin can regulate mitochondrial function enhance ATP production and reduce ROS thereby mitigating cellular damage and apoptosis. This effectively improves skeletal muscle atrophy (Wang et al., 2022).

#### 3.1.2 Anthraquinone

Anthraquinone is a type of quinone compound found in the metabolic products of higher plants such as Polygonaceae, Fabaceae and Rubiaceae, and in lower plants like lichens, as well as in fungi. Modern research indicates that anthraquinone has anti-inflammatory (Panthong et al., 2020), antioxidant (Zhao and Zheng, 2023) and anti-cancer effects (Shen et al., 2024).

In the early stages of tumorigenesis, autophagy exerts a tumor-suppressive effect by clearing damaged organelles and maintaining genomic stability. During this phase, moderate activation of autophagy by anthraquinones contributes to the prevention of tumor development.

Emodin is a quinone anthracene substance extracted from rhubarb, a traditional Chinese medicine. It is also a quinone anthracene derivative that has been studied deeply at present. It has anti-inflammatory, anti-tumor and liver protection activities (Zhang et al., 2022). Qin et al. found that emodin can inhibit the growth of HepG2 cells in a dose - and time-dependent manner induce cell S phase and G2/M phase stagnation promote apoptosis of HepG2 cells and thus control the migration and invasion of HepG2 cells. It can be concluded that emodin can inhibit the growth and metastasis of hepatocellular carcinoma cells by regulating autophagy-mediated degradation of snail and β-catenin (Qin et al., 2022).

In the advanced stages of tumor progression, cancer cells can utilize autophagy to resist nutritional deficiency or chemotherapeutic stress. At this point, autophagy induced by anthraquinones manifests as a protective response in tumor cells. The anthraquinone derivative C2, while resensitizing HCT116/L-OHP resistant cells to oxaliplatin and inducing apoptosis, also triggers a protective autophagy pathway in the tumor cells (Li et al., 2024). This finding indicates that even in the context of inhibiting tumor growth, anthraquinone compounds can still induce protective autophagy in tumor cells. This also implies that during the advanced stages of tumor progression, treatment with anthraquinone compounds should be combined with autophagy inhibitors to achieve optimal therapeutic effects.

Anthraquinone and its derivatives are rich in structural diversity and can be targeted to induce cellular autophagy by regulating different upstream signaling pathways which has broad application prospects in the treatment of cancer through the regulating cellular autophagy. Quinones and anthracene derivatives can also exert organ protective effects by inducing protective autophagy. For example, Liu et al. found that rhodopsin attenuates epithelial mesenchymal transition during renal fibrosis by initiating cellular autophagy via BMP-7 thereby improving renal dysfunction and reducing pathological damage to the kidney by renal fibrosis (Liu et al., 2021). Emodin showed cytotoxicity by promoting apoptosis in the L02 human hepatocyte cell line and hepatoprotective effects were achieved by inhibiting autophagy induced by the PI3K/AKT/mTOR signaling pathway (Zheng et al., 2019). Quinones exert organ-protective effects by regulating autophagy and may inhibit organ damage from various diseases.

**Figure 1:**
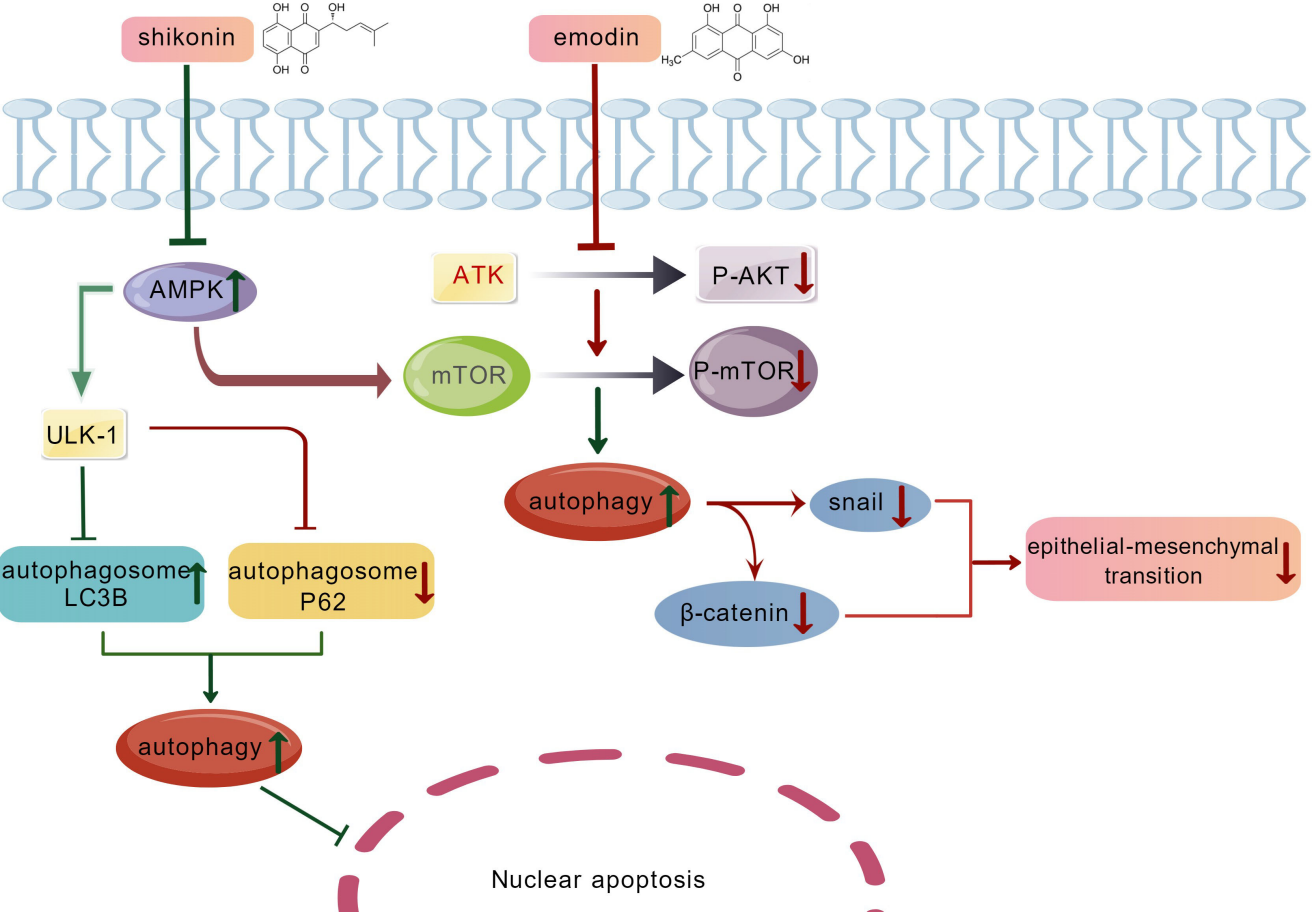
Quinone anthracene derivatives treat inflammation via the AMPK/mTOR/ULK-1 and AKT/mTOR pathways. (Green, activation; Red, inhibition.).

Quinone anthraquinone can inhibit the progression of certain inflammatory diseases by regulating autophagy in cells. For example, rhubarb induces apoptosis in fibroblasts from patients with ankylosing spondylitis (AS) by increasing the activity of caspase-9 and caspase-3 while simultaneously elevating Bax levels and downregulating Bcl-2. Additionally, rhubarb enhances autophagic activity in fibroblasts by upregulating the expression levels of Atg12 Atg5 and Beclin 1 (Ma et al., 2019). Shikonin can induce autophagy and apoptosis of fibroblast-like synoviocytes in rats with arthritis by regulating the AMPK/mTOR/ULK-1 pathway thereby inhibiting the further development of rheumatoid arthritis (Wang et al., 2024). Quinanthraquinone compounds can regulate autophagy to inhibit the development of arthritis both *in vivo* and *in vitro* by modulating upstream signaling pathways which may provide new insights for the treatment of arthritis (**Figure 1**).

Conjointly, according to modern pharmacological studies quinone anthracene derivatives have significant efficacy in the treatment of acute myeloid leukemia (Chen et al., 2014), renal fibrosis (Lu et al., 2025), osteoarthrosis (Zhang et al., 2025), atherosclerosis (Wani et al., 2025) and cancer (Li et al., 2024). As a plant-derived compound, quinone anthracene offers distinct advantages including wide availability, low cost, and demonstrated safety—no serious side effects have been observed in long-term clinical use. Owing to its simple extraction process and favorable profile, this compound may emerge as a promising new adjuvant therapy.

#### 3.1.3 Coumarin

Coumarins are found in higher plants of the Umbelliferae and Rutaceae families such as cinnamon cassia and lavender, which have unique biological activities in antibacterial (Ingole et al., 2025), anti-tumor (Qu et al., 2025), anti-viral (Wang et al., 2017), anti-inflammatory (Zhou et al., 2025), and anti-oxidant (Li et al., 2020). Coumarins have a wide range of biological activities by interacting with various enzymes and receptors in living organisms. Recent studies have demonstrated that coumarins have great potential for the treatment of certain diseases.

The anticancer effects of coumarins and their derivatives have been previously demonstrated, and one of the anticancer mechanisms of coumarins is the control of cancer cell proliferation or metastasis through the regulation of cancer cell autophagy. A novel coumarin derivative with a phenylsulfonylfuroxan moiety inhibits lung adenocarcinoma cell proliferation by promoting autophagosome formation inducing upregulation of the expression of the cellular autophagy-associated protein LC3-II and increasing autophagic fluxes. This novel coumarin derivative with a phenylsulfonylfuroxan moiety induces autophagy in lung adenocarcinoma cells via Akt/mTOR and Erk signaling pathways (Wang et al., 2018). Novel hybrids of 3-benzyl coumarin seco-B ring derivatives and nitric oxide (NO) donor phenylsulfonylfuroxan activate the PI3K/AKT/mTOR signaling pathway leading to apoptosis and autophagy induced in non-small cell lung cancer cells (Dong et al., 2019). In contrast, there also some studies have found that patients treated with furanocoumarins may induce squamous cell carcinomas in the setting of UVA exposure (Nijsten and Stern, 2003).

Tumor cells can restore the activity of the PI3K/AKT/mTOR pathway through bypass activation, thereby evading the autophagy regulation by PSMs. However, iso-coumarins can exert anti-tumor effects by continuously inhibiting the PI3K/AKT/mTOR pathway (Aqib et al., 2025).

Current research on coumarins has identified their anticancer mechanisms through multiple signaling pathways across various cancer types. These compounds demonstrate therapeutic potential by inducing oxidative stress and apoptosis in cancer cells, while also by inhibiting proliferation, promoting autophagy-dependent cell death, and activating relevant hormone receptors.

Coumarins may decelerate aging or safeguard organs by modulating cellular autophagy. For instance, licorice-derived coumarin triggers autophagy in hepatocytes, facilitating the clearance of accumulated lipid droplets and damaged mitochondria while restoring endoplasmic reticulum homeostasis, thereby exerting hepatoprotective effects (Yan et al., 2018). Chamoli et al. found that mitophagy-inducing coumarin (MIC) prolonged the lifespan of *Caenorhabditis elegans* in an HLH-30/TFEB-dependent and mitophagy-dependent manner involving DCT-1/BNIP3 and also prevented mitochondrial dysfunction in mammalian cells (Chamoli et al., 2023). These studies could mean that coumarins could theoretically slow or even delay the progression of age-related diseases with implications for human health and life extension.

The antimicrobial capacity of coumarins has been demonstrated in previous studies. The natural coumarin daphnetin (DAPH) enhances the antibacterial activity of microphage against methicillin-resistant *Staphylococcus aureus* (MRSA) and suppresses inflammatory responses by activating the mTOR-dependent autophagic pathway (Zhang et al., 2019). This finding implies that coumarins may have important potential to treat infectious diseases, particularly that caused by antibiotic-resistant pathogens.

The therapeutic effects of coumarin in anti-inflammatory, antibacterial properties, and organ protection have been proven, and particularly in cancer treatment. However, research has identified coumarin and its derivatives as potential carcinogens (Nijsten and Stern, 2003). Regarding the application of coumarin in anticancer, it may be necessary to further study the toxicity mechanism of coumarin in order to control the carcinogenic effect of coumarin.

#### 3.1.4 Tannins

Tannin is a phenolic compound present in plant branches, leaves, fruits, seeds, and other parts. It has pharmacological effects such as hemostatic (Carnevale et al., 2014), antibacterial (Sahiner et al., 2025), anticancer (Hao et al., 2025), lowering blood pressure (Goh et al., 2023) and regulating immune function (Hu et al., 2024). In Chinese medicines, tannin-containing herbs, such as *Pentaphyllum*, *Hordeum vulgare*, *Rhubarb*, *Dioscorea* etc., are often used as astringents, antiemetic, antidiarrheal, and anticancer agents (Jing et al., 2022).

Based on the structure, natural tannins could be divided into two categories, hydrolyzable tannins, that can be easily hydrolyzed into monomers by acids and enzymes; and condensed tannins, which composed of flavonoid oligomers with different degrees of polymerization (Bhat et al., 1998). Both types of tannins have shown positive pharmacological properties for human health in experimental studies.

Tannins have now been shown to affect a variety of cell signaling pathways that inhibit cancer cell proliferation by inducing autophagy (Xiao et al., 2025), apoptosis (Ren et al., 2022), or stimulating cellular senescence mechanisms (Kwon et al., 2018). Proanthocyanidin B2 a tannin-like substance extracted from grape seeds is a potent antioxidant and free radical scavenger. It can induce gastric cancer cell autophagy by regulating the Akt/mTOR signaling pathway exerting anti-cancer cell proliferation and inducing cancer cell apoptosis (Li et al., 2021). Corilagin, a hydrolyzable tannin, significantly inhibits the proliferation of SGC7901 gastric cancer cells and BGC823 cells *in vitro*. Corilagin can induce autophagy in gastric cancer cells and increase the production of ROS in gastric cancer cells which can effectively inhibit the growth and proliferation of gastric cancer cells (Xu et al., 2019). Oligomeric procyanidins promote apoptosis in human glioblastoma U87 cells but only a few studies have demonstrated its anticancer potential in human glioblastoma cells (Zhang et al., 2010). In addition, proanthocyanidins can protect retinal pigment epithelial cells from high glucose-induced damage through the p53/mTOR autophagy pathway (Li et al., 2022a). As one of the most common derivatives of tannins, proanthocyanidins have a protective effect on dyslipidemia. Proanthocyanidins can regulate autophagic flux and significantly reduce blood lipid levels in mice obesity models (Shi et al., 2019). In the pathological state of Alzheimer’s disease, simply increasing autophagosome formation (inducing mitophagy) without sufficient lysosomal function can lead to impaired autophagic flux, thereby exacerbating pathological damage. Urolithin A, by normalizing lysosomal cathepsins, promotes autophagosome formation while simultaneously restoring lysosomal function, ensuring smooth autophagic flux, and thereby exerting neuroprotective effects (Hou et al., 2024). This indicates that the integrity of autophagic flux, rather than the level of autophagy activation alone, may determine the therapeutic direction of tannin compounds.

The results of the current study found that tannins and tannin derivatives can induce autophagy reactive oxygen species accumulation and even apoptosis in cancer cells in order to inhibit the proliferation and metastasis of cancer cells. At the same time, Tannins and their derivatives also have antidiabetic (Shi et al., 2019) and hypolipidemic (Li et al., 2022a) effects. It protects the body’s cells from damage caused by high blood sugar. Although some of the anti-cancer mechanisms of tannins have not been elucidated many studies have suggested that tannins have a more positive role in anti-cancer therapy. Tannins as natural products extracted from plants have the advantages of being easily accessible inexpensive and maintain low toxicity to human cells and can be intensively investigated as future anticancer agents or adjuvant anticancer agents. After treatment with the autophagy inhibitor chloroquine or knockdown of the key autophagy genes ATG5/Beclin-1, the anti-tumor proliferative and pro-apoptotic effects of Urolithin B were significantly inhibited(Dietary Urolithin B Suppresses Lung Tumorigenesis Correlating with Autophagy Induction and Gut Microbiota Remodeling (Sun et al., 2026). This means that induced autophagy is a necessary mechanism for tannin compounds to exert their anti-tumor effects. If tumor cells can evade autophagy induced by tannin compounds or upregulate protective autophagy as an escape mechanism, this may lead to therapeutic resistance to tannin compounds.

Tannins are also potent antioxidants and anti-inflammatory agents. Hydrolyzable tannin pomegranate glycerol, extracted from pomegranate juice, activates NF-κB and MAPK phosphorylation in macrophages and enhances their phagocytic capacity (Yin et al., 2024). It also modulates the Foxo1/Prg4/HIF3α axis in chondrocytes, thereby attenuates the progression of osteoarthritis (Liu et al., 2021). Based on the results of existing studies it can be hypothesized that tannins have some research value in the prevention and treatment of inflammatory diseases.

Tannins can also activate the NF-κB and MAPK pathways in HepG2.2.15 cells that contribute to the inhibition of hepatitis B virus (HBV) replication while inducing the onset of autophagy in HepG2.2.15 cells and effectively decreasing the levels of HBV DNA and viral antigens (HBsAg and HBeAg) in HepG2.2.15 cells (Wang et al., 2023). This finding implies that plant tannins can effectively inhibit HBV replication in cells perhaps as a novel anti-hepatitis B virus drug providing new research ideas for the clinical treatment of HBV as well as other viral infectious diseases.

Tannins are more widely used antioxidants and anticancer agents. Due to the inhibition of cancer cell proliferation through the modulation of cell signaling pathways and the low toxicity of tannins they may be used as primary or adjuvant drugs for the future treatment of cancer. In addition, the superior anti-inflammatory properties of tannins can also be studied in depth for the treatment of inflammatory diseases.

### 3.2 Terpenoids

Terpenoids are compounds and their derivatives formed by the head-to-tail connection of two or more isoprene units (C_58_). Based on the number of isoprene units, terpenoids can be classified into monoterpenes, sesquiterpenes, diterpenes, triterpenes, and polyterpenes, among others. Saponins, which are a special class of glycosides formed by the combination of terpenoids (and related steroidal compounds) with sugars, are also representative compounds of plant-derived terpenoid derivatives.

#### 3.2.1 Saponin

Saponins are steroidal or triterpenoidal glycoside compounds found in a wide variety of plants and highly abundant in many herbal medicines. Saponins are currently categorized into two groups triterpenoid saponins and steroidal saponins based on their chemical structure. The saponins extracted from plants have various pharmacological effects such as sedative (Wang et al., 2025), anti-inflammatory (Wang et al., 2025), cardiovascular protection (Ji et al., 2024) and antioxidant (Stefanowicz-Hajduk et al., 2024).

In myocardial myocardial ischemia/reperfusion injury (MIRI), activation of autophagy during the ischemic phase can clear damaged mitochondria, exerting a protective effect; however, excessive autophagy during the reperfusion phase may lead to cell death.

**Figure 2:**
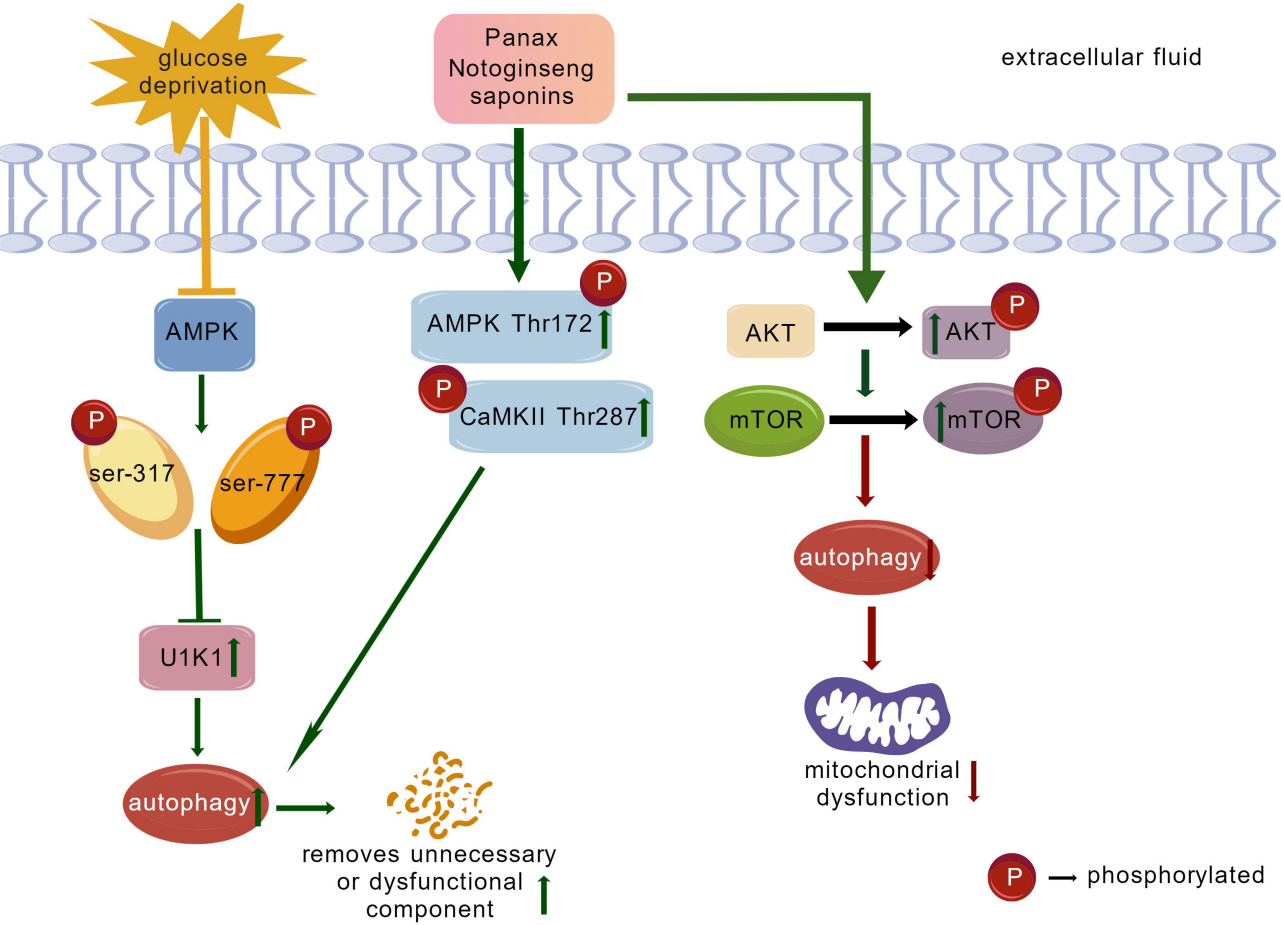
PNS induces cardiomyocyte autophagy to confer protection against acute myocardial infarction; PNS inhibits cardiomyocyte autophagy to provide protection against myocardial ischemia-reperfusion injury (MIRI). (Green, activation; Red, inhibition; Yellow, stress.).

Current research has demonstrated that plant-derived saponins can play a positive therapeutic role in cardiovascular disease by modulating cellular autophagy. Ginsenoside Rb1 inhibits cardiomyocyte autophagy by regulating the PI3K/Akt/mTOR signaling pathway, thereby exerting a protective effect against myocardial ischemia/reperfusion injury (MIRI) (Qin et al., 2021). *Panax notoginseng* saponin (PNS) extracted from the traditional Chinese medicine *Panax notoginseng* can enhance glucose deprivation (GD)-induced autophagy and thus exert cardioprotective effects by phosphorylating AMPK Thr172 and CaMKII Thr287 in cardiomyocytes (Wang et al., 2021). This finding demonstrates that PNS can protect cardiomyocytes in acute myocardial infarction and provides new theoretical support for the development of myocardial infarction drugs (**Figure 2**).

The anti-inflammatory effect of saponins is mainly achieved by inhibiting the activation of NF-κB, NF-AT and AP-1 signaling pathways and regulating cellular autophagy (Wong et al., 2009). For example, Ginsenoside Rh1 slows the progression of osteoarthritis in rats by regulating mitophagy through the AMPK/PINK1/Parkin pathway (Chen et al., 2025). Korean Red Ginseng saponin is anti-inflammatory by inhibiting the activation of NF-κB and AP-1 pathways in macrophages (Lee et al., 2022). Other studies have found that inhibition of TNF-α/NF-κB pathway activation by PNS R1 helps to reduce airway inflammation in a mouse model of asthma (Xue et al., 2020). As an anti-inflammatory agent with significant efficacy saponin has an undeniable potential in the treatment of inflammatory diseases.

In inflammatory diseases, saponin derivatives exert anti-inflammatory effects by regulating NF-κB signaling and autophagy pathways. During the acute inflammatory phase, inhibiting excessive cellular autophagy may help alleviate tissue damage; during the chronic inflammatory phase, restoring autophagic flux can clear damaged organelles and mitigate inflammatory progression.

Saponins are also a widely known anti-cancer agent. Ginsenoside Rk3 binds to PI3K/AKT and promotes autophagy and apoptosis in hepatocellular carcinoma cells inhibiting hepatocyte proliferation and development (Qu et al., 2023). Steroidal saponin A-24 extracted from *Allium sativum* induced autophagy and apoptosis in p53 wild-type gastric cancer cells by activating the PI3K/Akt/mTOR pathway and also activated the ROS-mTOR pathway in P53-deficient gastric cancer cells, thereby inducing autophagy and apoptosis in P53-deficient gastric cancer cells (Xu et al., 2021). It was found that saponins and their derivatives showed inhibition of the proliferation and spread of cancer cells in both *in vivo* and *in vitro* experiments. It elucidated the mechanism of action of saponin on inhibiting the proliferation of cancer cells inhibiting metastasis of cancer tissues and inducing autophagy and apoptosis of cancer cells and proved the role of saponin in inhibiting the development of cancer cells. Consistent with most PSMs, saponins activate autophagy in tumor cells during the early stages of tumorigenesis, which helps prevent tumor development. In the advanced stages of tumor progression, saponins induce protective autophagy in tumor cells, requiring combination with autophagy inhibitors to achieve optimal therapeutic efficacy.

Saikosaponin A selectively induces excessive autophagy in prostate cancer cells in the quiescent phase, while showing no significant cytotoxic effect on prostate cancer cells in the proliferative phase (Saikosaponin A enhances Docetaxel efficacy by selectively inducing death of dormant prostate cancer cells through excessive autophagy.). This indicates that the proliferative or quiescent state of cells can influence their sensitivity to saponin-induced autophagy.

Additionally, saponins also protect the relevant cells from oxidative stress damage by regulating cellular autophagy or cellular mitochondrial autophagy. In the prevention or treatment of chronic diseases such as diabetes (Yao et al., 2025) and fatty liver disease (Huang et al., 2017), saponins may be able to reduce the damage of the relevant organs while inhibiting the further progression of the disease.

### 3.3 Nitrogen-containing compounds

Plant-derived nitrogen-containing compounds are the most structurally diverse and biologically significant class of plant secondary metabolites. In phytochemical taxonomy, they constitute one of the three main classes alongside phenolics and terpenoids. Among these, alkaloids, which possess significant physiological activities and medicinal value, have been relatively more extensively studied.

#### 3.3.1 Alkaloid

Alkaloids are secondary metabolites found widely in plants and are organic compounds with cyclic structures containing one or more basic carbon atoms (Bhambhani et al., 2021). The exceptional therapeutic efficacy of alkaloids in the global arena has ignited a significant rise in research interest in their applications in analgesic, anti-inflammatory, anti-endotoxin, anti-tumor and other aspects.

Growing evidence suggests that plant-derived alkaloids can induce apoptosis in cancer cells through cytotoxicity and also inhibit cancer cell proliferation through activation of autophagy signaling pathway. Sophflarine A (SFA), a picrasidine derivative from the Traditional Chinese Medicine *Sophora flavescens*, promotes non-small cell lung cancer (NSCLC) cell death by inducing cellular pyroptosis through activation of the NLRP3/caspase-1/GSDMD signaling pathway and inhibits cancer cell proliferation by increasing ROS production to activate autophagy through blocking the PI3K/AKT/mTOR signaling pathway (Luo et al., 2023). Evodiamine induces intracellular calcium/JNK signaling-mediated autophagy and calcium/mitochondria-mediated apoptosis in glioma cells (Liu et al., 2013). Bisindolylmaleimide alkaloid 155Cl (BMA-155Cl) activates the NF-κB p65 pathway involved in cellular autophagy and is able to induce autophagy and apoptosis in human hepatocellular carcinoma HepG-2 cells *in vitro* and *in vivo* (Sun et al., 2017). Recent studies have found that tetrandrine can inhibit the phosphorylation of AKT and mTOR, downregulate Bcl-2 expression, upregulate Beclin-1 expression, and pro-mote the transcription and protein expression of ATG7 in pancreatic cancer cells. However, the autophagy induced by tetrandrine has been demonstrated to be cytoprotective. Studies have found that when combined with the autophagy inhibitor or with the silencing of the ATG7 gene, the autophagic process is blocked, leading to an accumulation of ROS, a decrease in mitochondrial membrane potential, and a significant increase in the apoptosis rate (Wang et al., 2024). When autophagy is activated but the degradation stage is impeded, a large number of immature autophagosomes accumulate within the cell, which in itself exerts a significant detrimental effect on the cells. This finding has important clinical implications: the autophagy induced by PSMs is inherently a protective response; however, if it is converted into an “obstructed type” (i.e., combined with an autophagy inhibitor), a functional switch from cytoprotection to cytotoxicity can be achieved, significantly enhancing antitumor efficacy.

Recently, it has been recognized that alkaloids and their derivatives may possess cardioprotective properties. In a rat model of spontaneous hypertension, Rhynchophylla Total Alkaloid (RTA) improves endothelium-dependent vasodilatory function by regulating cellular autophagy involved in the AMPK signaling pathway and also by enhancing the antioxidant capacity of vascular endothelial cells and attenuating oxidative damage (Li et al., 2018). These studies shown that alkaloids have great potential for the treatment of cardiovascular diseases by regulating the cellular autophagy pathway and that plant alkaloids and their derivatives can improve vascular function and cardiomyocyte function to play a cardioprotective role and can be used as a novel therapeutic tool in the treatment of chronic cardiovascular diseases.

Alkaloids can ameliorate the inflammatory response by modulating cellular autophagy to reduce the levels of relevant inflammatory factors in biological tissues. For example, by activating PINK1/Parkin-mediated mitochondrial autophagy to alleviate chondrocyte inflammation and halt the progression of osteoarthritis in rats (Kong et al., 2024). Certain alkaloids and their derivatives ameliorate neurodegenerative diseases through autophagy activation. In a mouse model of SNCA-induced Parkinson’s disease (PD) piperine activates P2RX4 to promote the fusion of lysosomal membranes with autophagosomes and activates autophagic flux in neuronal cells which subsequently leads to the degradation of pathological SNCA. This means that piperine may be an effective drug for Parkinson’s disease (Li et al., 2022b). The mechanism of action of alkaloids in inflammatory response and neuroprotection needs to be validated by further studies but the current study proves that alkaloids and their derivatives have a theoretical basis for in-depth research in neurotherapy. In the early stages of neurodegenerative diseases, when the autophagic function of neurons is not yet fully impaired, quinone-anthracene derivatives are more effective in restoring autophagic flux, playing a positive role in disease treatment. However, as the disease progresses and a large number of neurons die, the regulatory effect of autophagy becomes limited. At this stage, the therapeutic outcomes of using alkaloid derivatives to induce autophagy for treating neurodegenerative diseases are not ideal.

It is worth noting that most plant alkaloids and their extracts exhibit a degree of cytotoxicity, which can pose risks to living organisms. Nevertheless, in the treatment of certain diseases—such as cancer—these alkaloids and their derivatives hold significant research value that should not be overlooked. Balancing the therapeutic potential of alkaloids with effective control of their toxic effects remains a critical challenge for current research.

### 3.4 Carbohydrates

Plant-derived carbohydrates are the most abundant class of organic compounds in plants, primarily formed by the connection of monosaccharides through glycosidic bonds. Carbohydrates serve as both primary metabolites and include bioactive secondary metabolites. Plant polysaccharides with pharmacological activity are important substances among carbohydrate compounds that regulate cellular autophagy.

#### 3.4.1 Polysaccharide

Polysaccharides are naturally occurring macromolecular polymers usually consisting of more than a dozen monosaccharides bonded by straight or branched glycosidic chains. It is mainly found in plants, animals, microorganisms, and algae, as an important component of the cell wall. Polysaccharides as one of the important components involved in biological life activities play an important role in intercellular communication (Sheng et al., 2007), cell adhesion (Jiang et al., 2014), and molecular recognition in the immune system (Bürgel et al., 2025).In recent years, polysaccharides isolated from plants have received widespread attention because of their various pharmacological effects, such as antioxidant, antitumor, immunomodulatory, and anti-inflammatory effects. Since polysaccharides are macromolecular compounds with limited oral bioavailability, their application scope is relatively limited at present.

Polysaccharide extracted from the roots of *Echinacea purpurea* modulates hepatocyte autophagy to attenuate inflammation and hepatocyte apoptosis induced by oxidative stress-induced hepatic injury (Yu et al., 2022). Echinacea polysaccharide shows significant hepatoprotective effect on acetaminophen-induced drug-induced liver injury. Plant polysaccharides attenuate cellular inflammation and slow down apoptosis of damaged cells by regulating cellular autophagy and related signaling pathways. Plant polysaccharides could be a novel option for anti-inflammatory therapy.

The antioxidant effects of plant polysaccharides have been demonstrated in previous studies but the specific antioxidant pathways remain under investigation. Angelica polysaccharide attenuate hypoxia-induced neural stem cell injury by maintaining cell viability and blocking apoptosis and autophagy through the activation of the mTOR and Notch signaling pathways and the down-regulation of the Bcl2/adenovirus EIB 19kD interaction protein 3 (BNIP3) (Xue et al., 2019). This implies that part of the antioxidant effect of polysaccharides is through modulation of cellular autophagy.

Polysaccharides derived from plants can also inhibit the growth and proliferation of cancer cells by activating the autophagy pathway. Water-soluble polysaccharides extracted from *Polygala tenuifolia* inhibit the proliferation of SPC-A-1 cells through the activation of FAS/FAS-L pathway-mediated cellular autophagy (Yu et al., 2020). This suggests that this water-soluble polysaccharide may inhibit the development of human lung adenocarcinoma. *Rosa rugosa* polysaccharide induces apoptosis in human cervical cancer cells by inducing autophagy mainly through activating the PI3K/AKT/mTOR pathway (Liu et al., 2022). Demonstrating the modulatory effects of plant polysaccharides on cellular autophagy and immunity plant polysaccharides alone or in combination with conventional cancer therapies for the treatment of related cancers show great potential for medical research.

In addition, polysaccharides can induce autophagy through AMPK phosphorylation, reducing the accumulation of α-synuclein in neuronal cells, which may hold certain application prospects in the prevention and treatment of neurodegenerative diseases (Gopar-Cuevas et al., 2023). It is currently believed that the mechanism of action of polysaccharides in the prevention and treatment of neurodegenerative diseases is closely associated with their regulation of autophagy in neuronal cells, and the AMPK autophagy pathway can be selected to further validate its mechanism of action.

Current studies have demonstrated that plant polysaccharides can exert a protective effect against cognitively impaired diseases by inhibiting inflammatory responses modulating cellular autophagy and protecting nerves. Plant polysaccharides are expected to be novel drugs for the treatment of various cognitive dysfunctional diseases.

## 4 Concluding remarks and prospects

**Figure 3:**
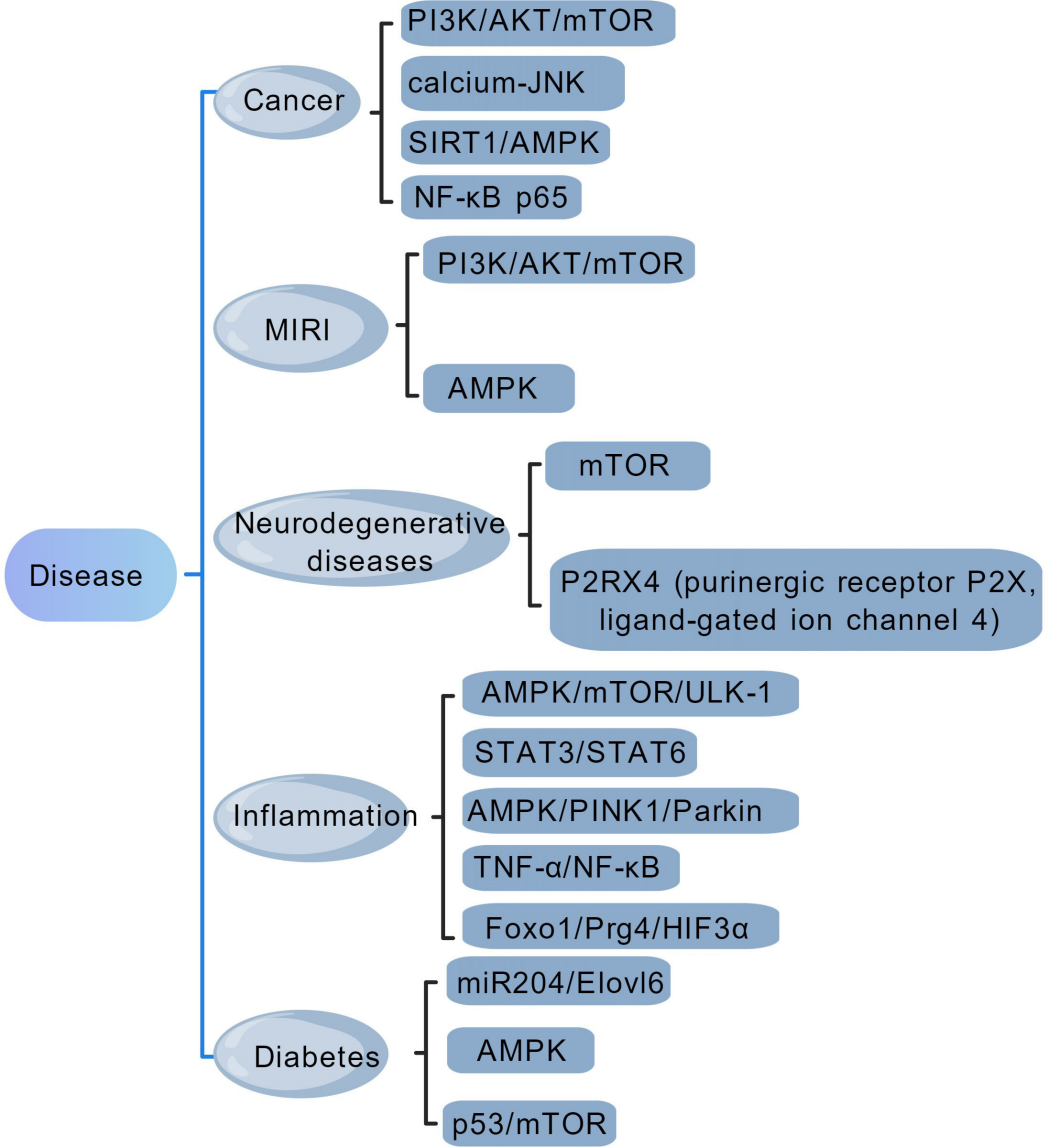
The primary therapeutic targets of plant secondary metabolites in autophagy-mediated disease treatment.

This article reviews the latest research advancements in recent years regarding various categories of plant secondary metabolites in the treatment of related diseases through the regulation of autophagy, and summarizes the key targets through which plant secondary metabolites modulate cellular autophagy to treat related diseases (**Figure 3**). The aim is to provide insights into the pharmacological applications of plant secondary metabolites that may modulate autophagy for the treatment of human diseases.

However, Current research on the regulation of autophagy by PSMs is primarily concentrated in the preclinical stage, lacking large-scale randomized controlled clinical trials. Most studies have not used autophagy as a primary outcome measure, limiting the clinical validation of their autophagy-regulating mechanisms. Current research often relies on the detection of static markers such as LC3-II and p62, lacking dynamic assessment of autophagic flux. The accumulation of autophagosomes and the enhancement of autophagic flux have profoundly different pathophysiological implications, yet existing detection methods are difficult to distinguish between them. Currently, most studies on PSMs focus on efficacy validation, while systematic evaluations of chronic toxicity, reproductive toxicity, carcinogenicity, and other aspects are insufficient. There is debate regarding the low bioavailability of most plant secondary metabolites *in vivo* and whether effective concentrations can be achieved *in vivo*. After the absorption and metabolism of polyphenolic compounds, the concentration of active metabolites that actually reach the target tissue may be insufficient to exert a therapeutic effect.

The activation of protective autophagy serves both as a therapeutic mechanism of PSMs and as a major source of risk for treatment resistance. Disease stage specificity further adds complexity to treatment strategies: In the early stages of tumors, activating autophagy can exert inhibitory effects, whereas in advanced stages, inhibiting protective autophagy is necessary. For metabolic diseases, restoring autophagic flux is crucial in the early stages, while combination with other therapeutic approaches is required in late stages. Future research should focus on elucidating the context-dependent mechanisms by which PSMs regulate autophagy, developing combination therapeutic strategies to overcome resistance risks, and promoting the clinical translation of PSMs as autophagy modulators by improving bioavailability and strengthening the chain of clinical evidence.

In the future, multi-omics approaches could be utilized to elucidate how factors such as PSMs concentration, timing, cell type, and microenvironmental conditions determine the direction of autophagic effects, thereby establishing personalized treatment strategies based on disease stage and molecular subtyping. A combination of multiple techniques, including multi-timepoint dynamic monitoring, autophagic flux analysis, and lysosomal function assessment, should be employed to accurately evaluate the true impact of PSMs on autophagy.

Furthermore, we look forward to witnessing more new developments in the modulation of autophagy by plant secondary metabolites for disease treatment as well as uncovering more intricate mechanisms underlying autophagy regulation.
